# How Does the Preparation of Rye Porridge Affect Molecular Weight Distribution of Extractable Dietary Fibers?

**DOI:** 10.3390/ijms12053381

**Published:** 2011-05-24

**Authors:** Allah Rakha, Per Åman, Roger Andersson

**Affiliations:** Department of Food Science, Swedish University of Agricultural Sciences, Box, 7051, S-75007 Uppsala, Sweden; E-Mails: Per.Aman@slu.se (P.A.); Roger.Andersson@slu.se (R.A.)

**Keywords:** whole grain rye, porridge making, arabinoxylan, fructan, β-glucan, molecular weight

## Abstract

Extractable dietary fiber (DF) plays an important role in nutrition. This study on porridge making with whole grain rye investigated the effect of rest time of flour slurries at room temperature before cooking and amount of flour and salt in the recipe on the content of DF components and molecular weight distribution of extractable fructan, mixed linkage (1→3)(1→4)-β-d-glucan (β-glucan) and arabinoxylan (AX) in the porridge. The content of total DF was increased (from about 20% to 23% of dry matter) during porridge making due to formation of insoluble resistant starch. A small but significant increase in the extractability of β-glucan (*P* = 0.016) and AX (*P* = 0.002) due to rest time was also noted. The molecular weight of extractable fructan and AX remained stable during porridge making. However, incubation of the rye flour slurries at increased temperature resulted in a significant decrease in extractable AX molecular weight. The molecular weight of extractable β-glucan decreased greatly during a rest time before cooking, most likely by the action of endogenous enzymes. The amount of salt and flour used in the recipe had small but significant effects on the molecular weight of β-glucan. These results show that whole grain rye porridge made without a rest time before cooking contains extractable DF components maintaining high molecular weights. High molecular weight is most likely of nutritional importance.

## 1. Introduction

Rye (*Secale cereale* L.) is an important cereal crop in Eastern and Northern Europe and a rich source of soluble and insoluble dietary fiber (DF) [1]. The typical rye grain contains 18 to 22% DF [2] or 14 to 21% when fructan is not included in the analysis [3]. The major components of rye DF are arabinoxylan (AX), fructan, and mixed linkage (1→3)(1→4)-β-d-glucan (β-glucan), with contents ranging from 8.0–12.1%, 4.5–6.4%, and 1.3–2.2%, respectively. The water soluble/extractable part of DF mainly comprises fructan and some AX and β-glucan, while the majority of the insoluble part consists of β-glucan and AX, as well as cellulose, Klason lignin, and small amounts of other polymers [2].

Cereal AX is a diverse group of polysaccharides based on a linear chain of 4-linked-β-d-xylopyranosyl residues substituted at O-2 and/or O-3 positions with α-l-arabinofuranosyl residues [4]. The polymer may also contain other sugar residues and phenolic substituents. Cereal β-glucan consists of about 70% 4-linked and 30% 3-linked β-d-glucopyranosyl residues, with some differences between species [5]. Fructan is a highly soluble DF component with prebiotic properties [6]. Chemically, fructan consists of β-linked oligomers and polymers of d-fructose with a terminal sucrose residue [7].

The European Food Safety Authority dietary guidelines (EFSA-Q-2008–467) recommend at least 25 g day^−1^ intake of DF for adults, while the typical Western diet gives less than 20 g DF day^−1^ [8]. Food authorities in many countries therefore recommend an increased intake of DF, and rye is the cereal with the highest DF content as shown in the database of United States Department of Agriculture [9]. The major benefits associated with DF are stool bulking effects, increased satiety, and reduced risk of developing type 2 diabetes and coronary heart disease [10,11]. Both the quantity and quality of the DF components are probably of importance in governing these physiological functions and health effects [12].

In the Nordic countries, rye is often consumed in the form of traditional whole grain soft breads and crispbreads, porridge and breakfast cereals. Porridge making is an easy and traditional way to prepare cereals for consumption, as it does not involve rigorous processing and the product retains its β-glucan molecular weight and viscosity better than bread or pasta [13,14]. It is known that the appearance of porridge differs depending on whether the cereal is added to cold or hot water before cooking. This difference may be at least partly related to changes in properties of the soluble DF components. Processing parameters, such as heating, mixing, and fermentation, have been reported to influence the content, structure, solubility, and properties of cereal DF [15–18]. Furthermore, endogenous and contaminating microbial enzymes present in the flour become active after hydration and can modify the DF components [15,17]. The activity of endogenous enzymes in rye grains is known to reduce extract viscosity [19] and influence the baking process and the quality of the bread [20].

Hence, knowledge of processing and enzyme-related modifications of cereal DF is of great importance. The main aim of this study was to examine potential changes in the molecular weight distribution of major extractable DF components (arabinoxylan, fructan, and β-glucan) during preparation of rye porridge.

## 2. Results and Discussion

### 2.1. Content of Dietary Fiber and Its Components

**Figure 1.**
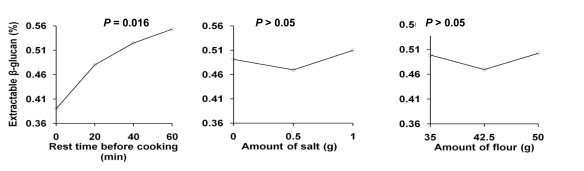

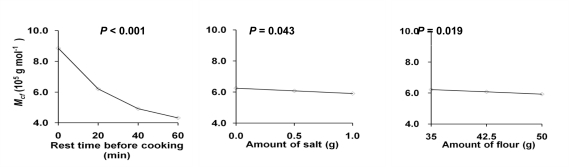
Main effects plot for extractable β-glucan (% of dry matter) and Calcofluor average molecular weight of β-glucan (*M_cf_*) in porridge.

The DF content of the whole grain rye flour used was about 20%, which is similar to previously reported values [2]. As a result of porridge making, the DF content increased to more than 23% (Table 1), most likely due to formation of resistant starch [21]. The total contents of DF, AX, β-glucan, and fructan in the two porridges were similar and not significantly affected by rest time before cooking. However, a small but statistically significant (*P* = 0.002) increase in the content of extractable AX was noted over the 60 min rest time. The extractable β-glucan content was 0.39% at 0 min rest time and increased by 41% to 0.55% (*P* = 0.016) after 60 min of rest time (Figure 1). These results clearly show that these dietary fiber components had a higher solubility after resting and that they were not hydrolyzed to small fragments or degraded to components not included in the content analyses. Increased solubilization of AX and β-glucan due to endogenous enzymes has also been reported during bread making [15,17,22,23]. Amount of salt or flour did not affect β-glucan extractability significantly.

### 2.2. Molecular Weight Distribution of DF Components

Measurement of molecular weight is a more sensitive way to assess process-induced changes in DF components. The molecular weight distribution of three major DF components, *i.e.*, fructan, β-glucan, and AX, in whole grain rye flour and porridges is described in the following sections.

#### 2.2.1. Molecular Weight Distribution of Fructan

Pre-trials were conducted to examine the effect of heating on the molecular weight distribution of fructan, since one of the aims of this study was to get an overview of fructan degradation during porridge making. Interestingly, there were no visible effects of increasing the cooking time (2, 10, 20 min) on the distribution of fructan with different degrees of polymerization (DP) (results not shown). Therefore cooking time was excluded as a factor in the experimental design.

**Figure 2.**
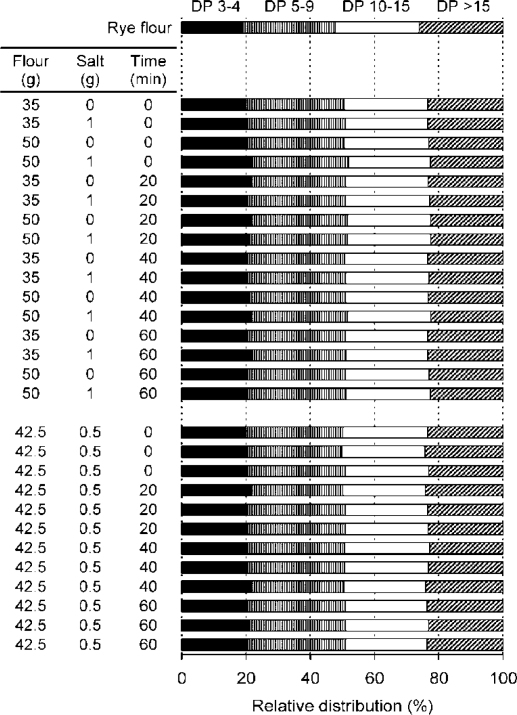
Relative distribution of fructan with different degrees of polymerization (DP) in whole grain rye flour and porridges prepared according to the experimental design. Results are computed from differences in peak areas of non-fructanase and fructanase treated extracts.

**Figure 3.**
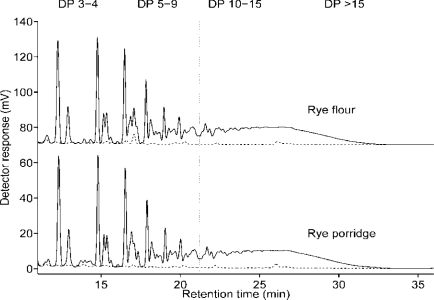
Chromatograms showing distribution of fructan in whole grain rye flour and porridge rested for 60 min before cooking. Dotted line (-------) shows chromatogram after fructanase treatment.

In the main experiment, the effects of salt, amount of flour and rest time of flour slurry before cooking on the relative DP distribution of fructan were non-significant (Figure 2). However, the rye flour had slightly more long-chain fructan compared with all porridges tested. The chromatograms for flour and porridge rested for 60 min were very similar (Figure 3). This indicates great stability of fructan during rye porridge making, and thus endogenous fructan hydrolases (FEHs), if present, appear to have very low activity. The DP distribution is only based on relative area and is thus likely to overestimate short oligomers, but this fact does not affect the conclusion that the fructan were stable. The complexity of linkages in cereal fructan and the specificity of FEHs might be the reason for the stability of fructan to endogenous enzymes in rye [24,25]. Another reason for the stability of fructan during resting in this study could be the sub-optimal temperature and pH (20 °C and 6.2, respectively), since the optimum temperature and pH for FEH activity have been shown to be around 40 °C and 4.5–5.5, respectively [26,27]. This stability of rye fructan to endogenous enzymes has also been observed during bread making, where breads made without yeast contain nearly as much fructan as the original rye grain [2,28]. This is an important consideration for modern processing of rye products, where the aim is to avoid losses of physiological functions associated with DF components.

#### 2.2.2. Molecular Weight Distributions of Arabinoxylan

**Figure 4.**
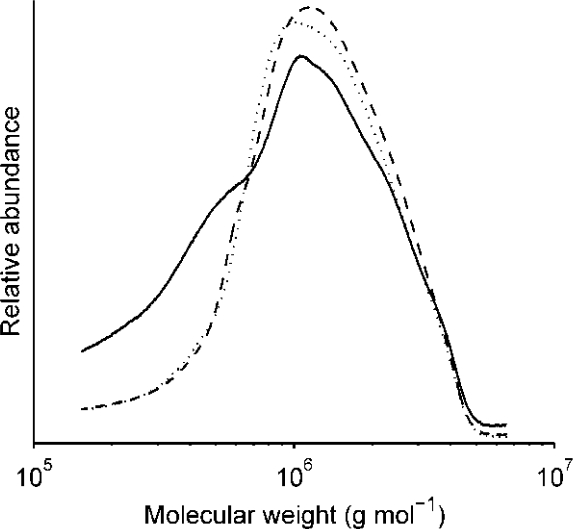
Molecular weight distribution of arabinoxylan in flour (——) and in porridges rested for 0 min (^_ _ _ _^) and 60 min (-------) before cooking.

**Figure 5.**
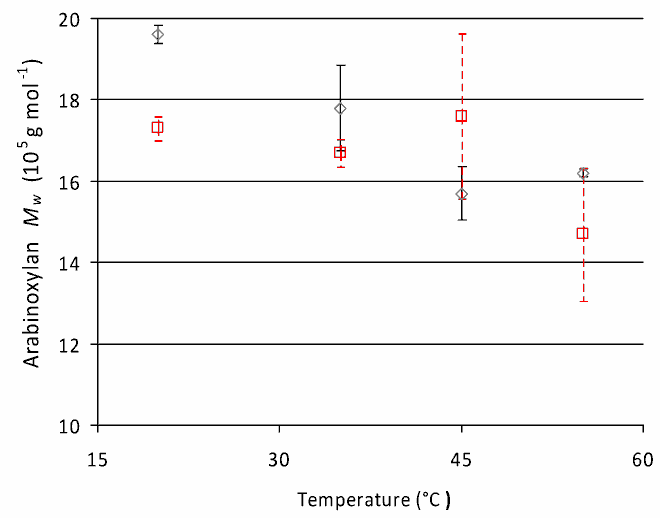
Effect of incubation temperature in rye flour slurries prepared with water (pH 6.2) and buffer (pH 4.5) on weight average molecular weight (*M_w_*) of rye arabinoxylan (⋄ pH 6.2; □ pH 4.5). The bars describe the standard error of mean (SEM).

Weight average molecular weight (*M_w_*) and number average molecular weight (*M_n_*) of extractable arabinoxylan were not significantly influenced by the amount of salt and flour in the recipe or the rest time during porridge making. This indicates the stability of arabinoxylan molecules during resting at 20 °C for 1 h and cooking of wholegrain rye porridge, as revealed by the molecular weight distribution profiles (Figure 4). The *M_w_* of AX in flour extract (12 × 10^5^ g mol^−1^) was slightly lower than in extracts of porridges (on average 14 × 10^5^ g mol^−1^) probably due to an increased extractability of high molecular weight fractions in porridge. Sub-optimal temperature (20 °C) and pH (6.2) during incubation were assumed to be responsible for non-existent activity of endogenous xylanases during the rest time. To test these hypotheses, rye flour slurries were incubated in an oven for 1 h at variable temperatures (20, 35, 45, 55 °C) and pH values (4.5 or 6.2) as described in experiment 2 of experimental section. The results from this investigation showed a significant decrease (*P* = 0.012) in AX *M_w_* as a result of increasing temperature, confirming our assumption about sub-optimal temperature during incubation being one possible reason for AX molecular weight stability (Figure 5). The optimum temperature for major rye AX-degrading enzymes, *i.e.*, *endo*-(1→4)-β-d-xylanase (EC 3.2.1.8), α-l-arabinosidase (EC 3.2.1.55), and β-d-xylosidase (EC 3.2.1.37), has been reported to be 40 °C, 60 °C and 70 °C, respectively, and the optimum pH around 4.5 [18]. However, the effect of pH on AX *M_w_* was non-significant (*P* > 0.05, Figure 5).

In contrast to porridge making, bread making normally involves dough proofing at 40–45 °C, which is the optimal temperature for yeast to work [29]. The dough proofing temperature is also ideal for *endo*-(1→4)-β-d-xylanase, which hydrolyses the β(1→4)-d-xylosidic linkage [18,19]. Furthermore, sourdough is used extensively in rye bread making to give the bread a characteristic flavor. The pH of sourdough (3.5–4.5) is usually closer to the optimum for endogenous xylanases [30], and relatively more AX degradation has been reported in sourdough bread compared with yeast or air-leavened crispbread [2].

Alongside *endo*-(1→4)-β-d-xylanase, α-l-arabinosidase liberates arabinose side-chains from the xylan backbone, while β-d-xylosidase attacks the non-reducing end of xylo-oligosaccharides and produces xylose monomers [19,31]. Other de-branching enzymes such as feruloyl esterase (EC 3.1.1.73), α-glucuronidase (3.2.1.139), and acetyl- and acetyl-xylan esterases (3.1.1.6 & 3.1.1.72) might be required for complete hydrolysis of AX, depending upon the complexity of branching on the xylan backbone [32]. The presence of multiple cross-linkages of AX with other polymers and side-chains ensures maximum stability. The extent of branching usually controls the solubility and enzyme action on AX molecules [4].

Xylanase activity may also arise from the microbial community present on the surface of cereal grains [17,33], and thus the activity is also determined by pre-processing of the grains before milling. The presence of *Triticum aestivum* xylanase inhibitor (TAXI) and xylanase inhibitor protein (XIP) along with some chemical inhibitors also contributes to the stability of AX in rye products [34,35]. The activity of endogenous enzymes and inhibitors depends on multiple factors, e.g., cultivar, growth conditions, pre-harvest sprouting, storage conditions, milling fractionation, and processing conditions [19,35].

#### 2.2.3. Molecular Weight of Extractable β-Glucan

Of the three major DF components studied here, β-glucan was the most sensitive to enzyme-related degradation. β-Glucan Calcofluor average molecular weight (*M_cf_*) was significantly influenced by rest time (*P* < 0.001), and amount of salt (*P* = 0.043) and flour (*P* = 0.019) in the recipe (Figure 1). For β-glucan there was a sharp decline in *M_cf_* from 0–20 min rest time, followed by a gradual decreasing trend. The average *M_cf_* value in porridges made without any rest time was 8.86 × 10^5^ g mol^−1^ and this decreased to 4.32 × 10^5^ g mol^−1^ after 60 min rest time before cooking. The inverse relationship observed here between *M_cf_* and extractability shows that extractable β-glucan is released from the insoluble matrix, while at the same time the molecular weight is decreased. Endogenous β-glucanases are probably responsible for these effects [22], since heat during cooking of different porridges has been shown not to affect the molecular weight of β-glucan [14,36]. A small but statistically significant decrease in *M_cf_* (from 6.25 × 10^5^ to 5.91 × 10^5^ g mol^−1^) was observed with addition of 1 g salt (Figure 1). This decrease in *M_cf_* might be due to increased activity of endogenous enzymes in the presence of salt. The *M_cf_* value also decreased somewhat with increasing flour amount (Figure 1). A possible reason for this decrease might be a change in water content affecting enzyme activity.

**Figure 6.**
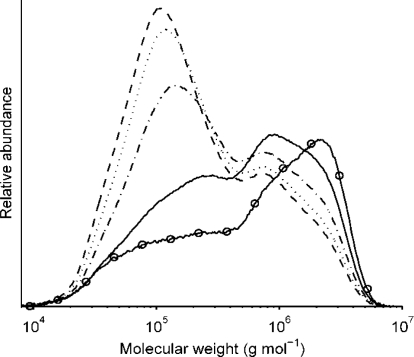
Molecular weight distribution of β-glucan in flour and in porridges rested for 0, 20, 40, 60 min before cooking ( 
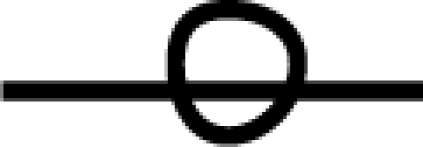
 flour, —— 0 min, – - – - – 20 min, ------- 40 min, _ _ _ _ 60 min).

The molecular weight distribution profiles of β-glucan were compared at each time interval at the centre point of flour and salt (Figure 6). The raw flour had the highest proportion of high molecular weight β-glucan fraction, while in porridges the low molecular weight fraction increased with increasing rest time before cooking. The porridges made without any rest time also showed significant degradation compared with flour. This is an indication that endogenous enzymes hydrolyze β-glucan rapidly and that the majority of this degradation takes place within the first few minutes, as also shown by Andersson *et al.* [22] in a bread study. Other processing parameters such as dough mixing have been shown to affect *M_cf_* significantly too [15]. Processing could therefore influence the structural and molecular properties of β-glucan. This enzyme-related degradation could be undesirable and may impair the nutritional value of β-glucan [12].

## 3. Experimental Section

### 3.1. Materials

Finely milled whole grain rye flour was purchased from a local supermarket and thoroughly mixed to produce homogeneous fractions. The salt (NaCl) used during porridge making was laboratory grade.

### 3.2. Chemicals and Reagents

Anhydrous sodium acetate was procured from Fluka (Steinheim, Germany). The enzymes used for analysis were obtained from Megazyme (Bray, Ireland). Lactose was obtained from Sigma (Steinheim, Germany).

### 3.3. Experiment 1

#### 3.3.1. Design of Experiment 1

A full factorial design was used, with two levels of flour (50 and 35 g) and salt (0 and 1 g) and four levels of rest time (0, 20, 40, 60 min) before cooking (Figure 2). The centre points of salt (0.5 g) and flour (42.5 g) with all four levels of rest time were carried out in triplicate to account for experimental error.

#### 3.3.2. Porridge Making

Pre-trials were conducted to determine the optimum amounts of flour and water and the optimum heating time to produce good, consistent porridge. The experimental porridges were then prepared according to the design using fixed amount (180 mL) of water. The water-soaked ingredients were incubated at 20 °C and stirred for 10 s every 5 min to ensure uniform action of the endogenous enzymes. After cooking for 7 min on a hot plate at intermediate setting, the porridges were transferred to an aluminum container, covered and immediately frozen at −20 °C. The next day the porridges were freeze-dried for 72 h. Finally, the freeze-dried porridges were milled in a cyclone sample mill (Retsch, Hann, Germany) to pass a 0.5 mm screen and stored in plastic containers at room temperature for subsequent analysis. This method of porridge making is very similar to that normally used in Scandinavia.

### 3.4. Experiment 2

This experiment was planned to investigate the role of temperature and pH in AX degradation. 10 g finely milled whole grain rye flour (different from the batch used in experiment 1) was weighed and 27 mL water or 0.5 M sodium acetate buffer was added. The rye flour slurries having pH 6.2 (prepared with water) and 4.5 (prepared with buffer) were incubated for 1 h at variable temperatures (20, 35, 45 and 55 °C) in an oven. The slurries were stirred for 10 s every 5 min to ensure uniform action of the endogenous enzymes. Subsequently the slurries were freeze dried and milled in a cyclone sample mill (Retsch, Hann, Germany) to pass a 0.5 mm screen.

### 3.5. Analytical Methods

All analytical results are reported on a dry matter basis. Analyses were replicated at least twice on each sample (raw flour and porridges) and the deviation between replicates was generally less than 5%. Dry matter was determined by drying the samples at 105 °C for 16 h. AX was extracted during enzymatic degradation of starch at 100 °C and 60 °C followed by purification of the extract with lichenase and pancreatin before precipitation of AX with ethanol [2]. AX weight-average molecular weight and number-average molecular weight were determined by high performance size exclusion chromatography coupled with multiple angle laser light scattering and refractive index detectors (Wyatt Technology, Santa Barbara, USA). Molecules with a molecular weight of less than 1.5 × 10^5^ g mol^−1^ were not included in the calculations, as they lack precision in this analysis. Fructan molecular weight distribution was investigated using high performance anion exchange chromatography with pulsed amperometric detection [28]. In brief, the samples were extracted with 80% ethanol in a boiling water bath for 1 h. The ethanol was evaporated and the residue was treated with amyloglucosidase (soluble starch 326 U mL^−1^, E-AMGDF, Megazyme, Bray, Ireland). The amyloglucosidase-treated solution was divided into two aliquots and one was treated with fructanase mixture, E-FRMXLQ (exo-inulinase 2000 U mL^−1^ and endo-inulinase 200 U mL^−1^, Megazyme, Bray, Ireland). Both aliquots were filtered through Titan2^®^ HPLC syringe filters (17 mm, 0.45 μm, Sun Sri, Wilmington, N.C, USA) before chromatography. The distribution was computed based on difference in peak area between non-fructanase and fructanase treated extracts since no reference compounds were available. Calcofluor average molecular weight of β-glucan was determined using size exclusion chromatography with Calcofluor detection according to the method described by Rimsten *et al.* [37], with a Calcofluor concentration of 0.0025%. With this detection technique, molecules smaller than 10^4^ g mol^−1^ are excluded from the analysis, as these shorter fragments cannot be quantified with precision.

Rye flour and selected porridges (with 42.5 g flour, 0.5 g salt and rested for 0 or 60 min) were analyzed for major DF components according to the Uppsala method [38], subsequently modified by Andersson *et al.* [39] for separate analysis of extractable and unextractable DF components. AX values were calculated from the arabinose, xylose, and galactose values, assuming an arabinose/galactose ratio of 0.69 in extractable arabinogalactan [40]. Fructan content was analyzed according to McCleary *et al.* [41] by a spectrophotometric method using the enzymatic assay kit K-FRUC (Megazyme, Bray, Ireland). The samples were treated with α-galactosidase from *Aspergillus niger* E-AGLAN (Megazyme, Bray, Ireland) before degradation of starch, maltosaccharides, and sucrose to avoid interference by raffinose series oligosaccharides. Total β-glucan was quantified by an enzymatic method [42], while extractable β-glucan was calculated by taking into account the area under the curve during molecular weight analysis, as described by Rimsten *et al.* [37].

### 3.6. Statistical Analyses

The responses from the designed experiment were analysed with regression (GLM procedure in Minitab 15 statistical software, Minitab Inc., State Collage, PA, USA) with factors defined as covariates. The contents of DF components were analysed by one-way ANOVA with the factor resting time. The effect of temperature on the molecular weight of arabinoxylan was evaluated by the GLM procedure with pH and temperature as factors with temperature defined as covariate.

## 4. Conclusions

This study showed that whole grain rye porridge is an excellent source of both extractable and unextractable DF. During porridge making, notable amounts of resistant starch (in the range of 3%) were formed, which increased the DF content to more than 23% of dry matter. The content of other major DF components such as AX, β-glucan, and fructan did not change significantly. However, the extractability of AX and especially β-glucan increased significantly when the flour slurry was allowed to rest before cooking. Molecular weight distribution profiles of extractable AX and fructan were remarkably stable in porridges prepared under different conditions, but the molecular weight of extractable β-glucan greatly decreased during a rest time before cooking. These results clearly show that porridge making is a useful way to prepare whole grain rye for human consumption, especially if prepared without rest-time before cooking, when the intention is to produce a meal with a high content of total DF and extractable DF with retained molecular weight.

## Figures and Tables

**Table 1.** Content (% dry matter) of dietary fiber (DF) and major DF components in rye flour and two porridges prepared with 42.5 g flour and 0.5 g salt and rested for 0 or 60 min before cooking. Means of double analysis of three experimental replicates, expressed on a dry matter basis ^1^.

**Sample**	**DF^2^**		**Arabinoxylan^3^**		**β-Glucan**		**Fructan**

	**Total**	**Extractable**	**Total**	**Extractable**	**Total**	**Extractable**	**Extractable**
Flour	20.2	7.5	8.2	2.5	2.2	0.31	4.2
Porridge, without rest time	23.2	7.3	8.6	2.5	2.3	0.39	3.9
Porridge, with 60 min rest time	23.5	7.7	8.6	2.6	2.3	0.55	4.1

1 The deviation in analytical results is generally below 5%;

2 Including fructan;

3 Calculated after subtracting arabinose residues in arabinogalactan, as described in materials and methods.
